# Transient Intermittent Hypoxia Exposure Disrupts Neonatal Bone Strength

**DOI:** 10.3389/fped.2016.00015

**Published:** 2016-03-07

**Authors:** Gyuyoup Kim, Omar Elnabawi, Daehwan Shin, Eung-Kwon Pae

**Affiliations:** ^1^Department of Orthodontics and Pediatric Dentistry, School of Dentistry, University of Maryland, Baltimore, MD, USA; ^2^BISCO, Inc., Schaumburg, IL, USA

**Keywords:** bone, hypoxia, neonates, osteoblast, zinc, ZIP8

## Abstract

A brief intermittent hypoxia (IH, ambient O_2_ levels alternating between room air and 12% O_2_) for 1 h immediately after birth resulted in pancreatic islet dysfunction associated with zinc deficiency as previously reported. We hypothesized that IH exposure modulates zinc homeostasis in bone as well, which leads to increased bone fragility. To test this hypothesis, we used neonatal rats and human osteoblasts (HObs). To examine IH influences on osteoblasts devoid of neural influences, we quantified amounts of alkaline phosphatase and mineralization in IH-treated HObs. Bones harvested from IH-treated animals showed significantly reduced hardness and elasticity. The IH group also showed discretely decreased levels of alkaline phosphatase and mineralization amounts. The IH group showed a decreased expression of ZIP8 or Zrt and Irt-like protein 8 (a zinc uptake transporter), Runx2 (or Runt-related transcription factor 2, a master protein in bone formation), Collagen-1 (a major protein comprising the extracellular matrix of the bone), osteocalcin, and zinc content. When zinc was eliminated from the media containing HObs using a zinc chelate and added later with zinc sulfate, Runx2, ZIP8, and osteocalcin expression decreased first, and recovered with zinc supplementation. Adenovirus-mediated ZIP8 over-expression in osteoblasts increased mineralization significantly as well. We conclude that IH impairs zinc homeostasis in bones and osteoblasts, and that such disturbances decrease bone strength, which can be recovered by zinc supplementation.

## Introduction

Chronic, repetitive, short-term exposure to hypoxia is associated with fragile bones in adults (1). However, reports of subnormal bone quality resulting from hypoxia in neonates or infants are rare. Short-term exposure to hypoxic events is common in prematurely born neonatal humans, principally in the form of apnea of infancy (2, 3). Apnea of prematurity, in particular, is a breathing pattern that results in intermittent hypoxia (IH) exposure, the repetitive presentation of short-term hypoxia followed by restoration of oxygen. IH exposure injures cardiovascular sympathetic ganglia (4), as well as the dorsal motor nucleus of the vagus (5, 6), and potentially elicits significant glucose and lipid dysregulation in later life (7, 8). Recently, our lab demonstrated diabetogenic effects resulting from brief IH exposure to rat neonates, including reduced zinc levels in pancreatic beta cells, contributing to lower insulin and high blood glucose levels (9, 10). Thus, we suspected that this short-term IH challenge could increase bone fragility potentially mediated by impaired zinc homeostasis. During IH exposure, oxidative stress, induced by re-oxygenation, may increase reactive oxygen species in the IH affected bone cells, which may favor osteoporosis as a consequence of oxidative stress that off-balances bone remodeling processes between osteoblastic and osteoclastic activities (11, 12).

Only sparse mechanistic clinical evidence is available on cause–effect relationships between IH challenges and reduced bone strength in human neonates. Type-2 diabetes is commonly comorbid with obstructive sleep apnea, a disease accompanied by IH, lending suspicion that shared processes operate in both the metabolic and breathing disorders (13–15). Conceivable causal mechanisms for increased bone fragility as sequelae of IH include glucose dysregulation (10, 16), increased sympathetic nervous system activity (17, 18), and proinflammatory conditions (19, 20) in addition to disrupted homeostasis of zinc, an essential trace metal in cell metabolism. We hypothesize that IH exposure in neonates may result in fragile bones *via* zinc deficiency in later life, irrespective of glucose metabolic dysfunction. Using a neonatal rat model developed in our lab and human osteoblast (HOb) cells, we evaluated whether a 1-h IH exposure during the neonatal period increases bone fragility and whether this fragility is associated with reduced zinc levels resulted from IH exposure.

## Materials and Methods

### Preparation of Animals

Near end-term pregnant Sprague-Dawley rats were purchased and maintained until parturition. Within 3 h after birth, 12 offspring (5 males and 7 females), together with the mother of this experimental group, were randomly placed in a customized IH chamber that alternated oxygen concentration between room air (20.5% O_2_) and a hypoxic condition (12% O_2_) every 240 s for 1 h, with food and water accessible *ad libitum*, as described previously (9, 21). Ten control pups (five males and five females) and their mother were maintained in room air. After the initial 1-h IH treatment, pups were kept in room air until euthanasia. To examine long-term IH effects on glucose regulation, we maintained IH-treated and untreated control pups for 12 weeks and measured their blood glucose and insulin levels. All procedures carried out and related to the procedure and animal care were approved by the Institutional Animal Care and Use Committee of the University of Maryland, Baltimore (Permit number D121101).

### Bone Physical Strength Studies

**Figure 1:**
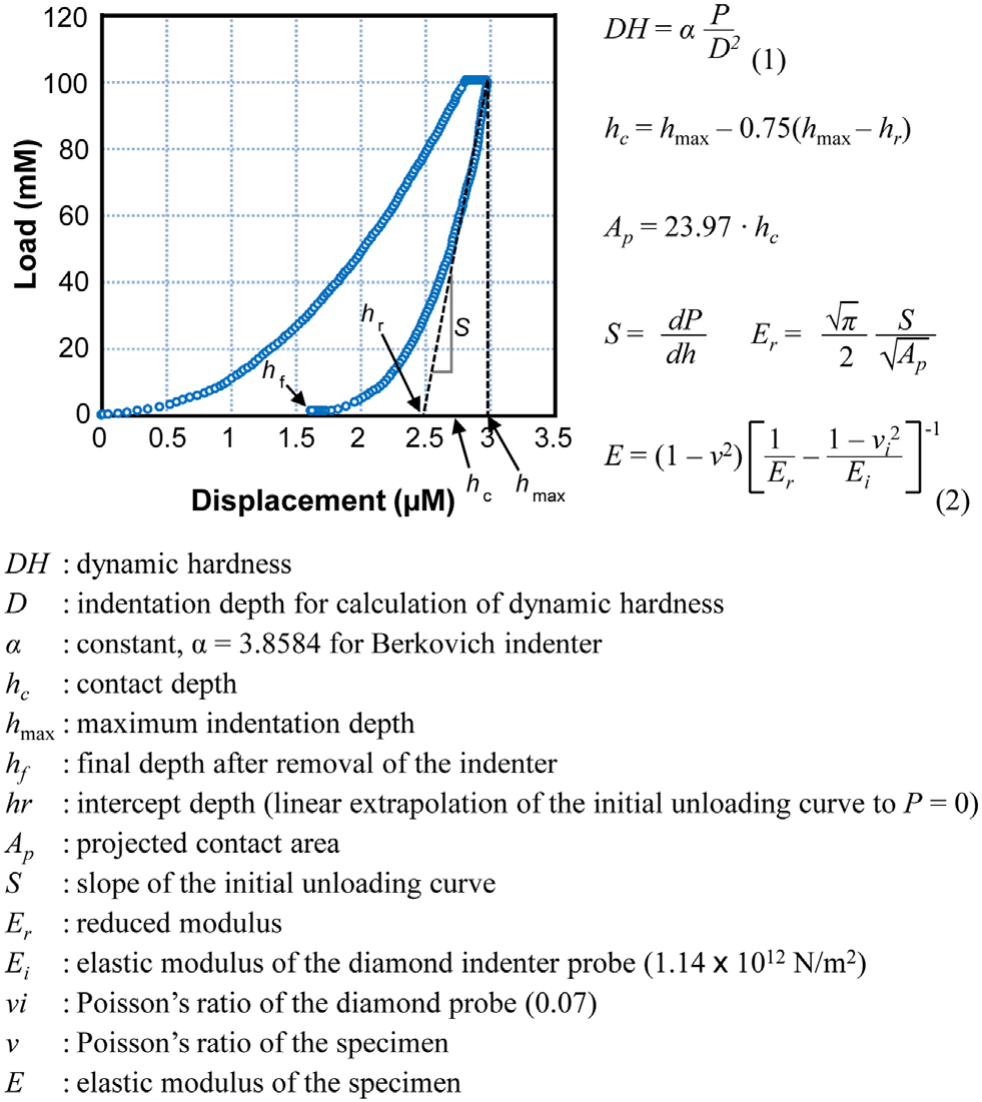
**A typical load–displacement curve generated by DUH-201S**. Dynamic hardness and elastic modulus were calculated using the equations presented. Dynamic hardness of a bone (or DH, see Eq. 1) was determined using test force (*P*) and indentation depth (*D*) during the indentation process. Reported dynamic hardness values were calculated at the end of indentation before retrieving the indenter. Elastic modulus of the sample (or *E*, see Eq. 2) was determined using the relationship of reduced modulus (*E_r_*), elastic modulus of the indenter (*E_i_*), and elastic modulus of the bone sample (*E*). Reduced modulus was obtained using the projected area of contact (*A*_P_) and elastic contact stiffness (*S*), which was measured from the slope of the initial portion of the unloading curve.

To estimate physical strength of bone, we used micro-hardness [dynamic hardness (DH)] and elastic modulus of the tibia and mandible. The bones were isolated from all 3-week-old rats and were measured using an ultra-micro hardness tester (DUH-W201S, Shimadzu, Japan). Each bone specimen was embedded in poly-(methyl methacrylate) (PMMA) mold, and the sample surface was ground and polished using grit paper. Maximum indentation force of 100 mN was applied onto the bone surface using a Berkovich indenter throughout the experiments. DH was calculated by measuring an indentation depth at the end of the indentation process before unloading and maximum indentation force. DH, indicated in millinewton per square micrometer, is a load-bearing material property of indentation contact, based on both plastic and elastic deformations of a specimen under test. As the indenter was driven into the surface of test samples, both elastic and plastic deformations occur. Since the DH measure is obtained during indentation loading, DH represents hardness of material based on both elastic and plastic deformations of bone. Calculated elastic modulus of a sample is based on elastic contact theory. Elastic modulus (*E*), indicated in gigapascal, was calculated from the initial portion of the elastic unloading curve, based on the method proposed by Oliver and Pharr (22) (Figure 1). This relationship was driven by the assumption that elastic displacements are produced by both the indenter and the specimen.

### Culturing Human Osteoblasts

Human osteoblasts (Cell Applications, Inc.) were maintained in normal osteoblast growth medium (OGM), with added fetal bovine serum, ascorbic acid, and gentamicin/amphotericin-B (Lonza Walkersville, Inc.), under standard conditions in a 37°C and 5% CO_2_ incubator. Differentiation and mineralization of osteoblasts were induced by supplementing OGM with 10 mM β-glycerophosphate and 200 nM hydrocortisone-21-hemisuccinate for 14 days. The medium was replaced every 2–3 days until cells were harvested.

Intermittent hypoxia treatment was performed and followed by cell differentiation. The purchased osteoblast cells were placed in a hypoxia chamber (Billups-Rothenberg, Inc.), and internal gases alternated between room air and a hypoxic gas mixture (1% O_2_, 5% CO_2_, and 94% N_2_) every 10 min, 10 times to mimic IH conditioning in tissue levels. After IH exposure, the osteoblasts were differentiated for 2 weeks. We could not maintain the cells for 3 weeks to match with our *in vivo* experiments due to cell senescence.

Zinc depletion/addition experiments were carried out concomitantly. At confluence, osteoblasts were treated with differentiation medium containing 5 μM TPEN [*N,N,N′,N′*-tetrakis-(2-pyridylmethyl)-ethylenediamine], which is a cell-permeable zinc-specific chelate, and followed by ZnSO_4_ treatment at 0, 10, and 50 μM concentrations for 14 days.

### Histological Staining

Additional 3-day-old male pups exposed to IH (*n* = 2) and room air (*n* = 2) conditions were euthanized using CO_2_ and fixed in 10% neutral buffered formalin (Sigma-Aldrich Co.) for 7 days. Hind limbs were isolated and outsourced to the Pathology and Laboratory Medicine Services at UCLA for immunohistochemistry staining. The specimens were decalcified for paraffin embedding. Comparable sections of paraffin embedded specimens were processed with anti-Runx2 antibody (Sigma-Aldrich Co.) on the slides in accordance with the manufacturer’s guidelines. All sections were visualized using diaminobenzidine reaction and counterstained with hematoxylin. The corresponding sections were stained with Masson Trichrome (Sigma-Aldrich Co.), in accordance with the manufacturer’s protocol. Histological evaluations were performed with Aperio ImageScope (Leica Microsystems, Inc.) on digital images.

### Western Blotting

Proteins were extracted from the harvested hind limbs of 3-week-old rats. Isolated femurs and tibias were snap-frozen by adding liquid nitrogen on a mortar and homogenized using a pestle. Pulverized samples were incubated in the red blood cell lysis buffer (155 mM NH_4_Cl, 140 mM NaHCO_3_, and 0.1 mM EDTA, pH 7.3) for 10 min, and supernatants were removed by centrifuge. Pellets were lysed in ice-cold RIPA buffer containing protease inhibitor cocktails (Roche Applied Science). A controlled amount of proteins was separated using SDS-PAGE and transferred onto a PVDF membrane using an electroblot. After blocking with 5% milk TBS-T, the blots were probed using primary antibodies (anti-ZIP8 antibody, Pierce Biotechnology, Inc.; anti-Runx2 antibody, Sigma-Aldrich Co.; anti-β-actin antibody, Cell Signaling Technology, Inc.; and anti-Collagen-1 antibody, Santa Cruz Biotech.) and followed by a horseradish peroxidase-conjugated secondary antibody. Chemiluminescent reagents were used to detect immunoreactive proteins, and the membrane was exposed to X-ray films.

### Zinc Assay

Intracellular zinc levels were estimated using Zinc Colorimetric Assay Kit (BioVision, Inc.) as previously described (9). Thirty micrograms of whole-cell lysate was precipitated by TCA, and the supernatant was mixed with the zinc reagent in the 96-well plate according to the manufacturer’s protocol. Absorbance was measured using Epoch spectrophotometer (BioTek Instruments, Inc.) at 560 nm.

### Alkaline Phosphatase Activity and Mineralization Assays

To estimate alkaline phosphatase activity, the harvested cells were washed with PBS and fixed for 1 min in 10% neutral buffered formalin. After fixation, the cells were washed with PBS and stained with BCIP–NBT substrate solution (Sigma-Aldrich Co.) for 10 min at room temperature, according to the manufacturer’s protocol. After staining, mono-layered cells were washed with PBST containing 0.05% Tween 20 and covered with PBS for visualization.

Alizarin Red S staining was performed to assess mineralization. The cells were fixed for 40 min in 10% neutral buffered formalin and washed with distilled water. The fixed cells were incubated with sufficient Alizarin Red S staining solution (Alfa Aesar) at room temperature in the dark for 45 min. After the solution was removed, the cell monolayer was washed with distilled water four times and PBS was added to visualize calcium deposits.

### ELISA Assay for Osteocalcin

Assays for intracellular osteocalcin quantification were performed with cell lysates using an ELISA kit (Takara Bio, Inc.), according to the manufacturer’s protocol. Shortly, samples were incubated on the monoclonal anti-osteocalcin coated plate for 2 h, followed by incubation with the peroxidase labeled antibody for 1 h. The peroxidase substrate 3,3′,5,5′-tetramethylbenzidine and stop solution were added for the reaction, developing a color. The amounts of osteocalcin were quantified *via* absorbance reading at 450 nm using a spectrophotometer.

### Adenoviral Vector of ZIP8

Adenovirus-GFP (as a control) and -ZIP8 (HA-tagged) were purchased from Applied Biological Materials, Inc. (Canada). At confluence, osteoblasts were infected with adenovirus for 36 h at a multiplicity of infection of 100, and followed by supplementing differentiation medium. Evaluation of adenoviral-ZIP8 infection was performed by Western blots using HA-probe (Santa Cruz Biotechnology, Inc.).

### Statistical Analysis

Statistical significance for group differences was tested by a two-tailed *t*-test. Each assay at cellular or subcellular level was repeated at least three times. Data were summarized in the form of mean ± SD for bone strength and elasticity measurements to exhibit a clear relationship between variances and sample size. Mean ± SEM was used for other measurements.

## Results

Outcomes of the analyses on the bones and HObs were compared between control and IH groups. Differences in gender were tested for bone physical strength only. Leg and mandibular bones harvested from the IH-treated rats and IH-treated HObs obtained *in vitro* showed parallel outcomes.

### Bone Hardness and Elasticity

**Figure 2:**
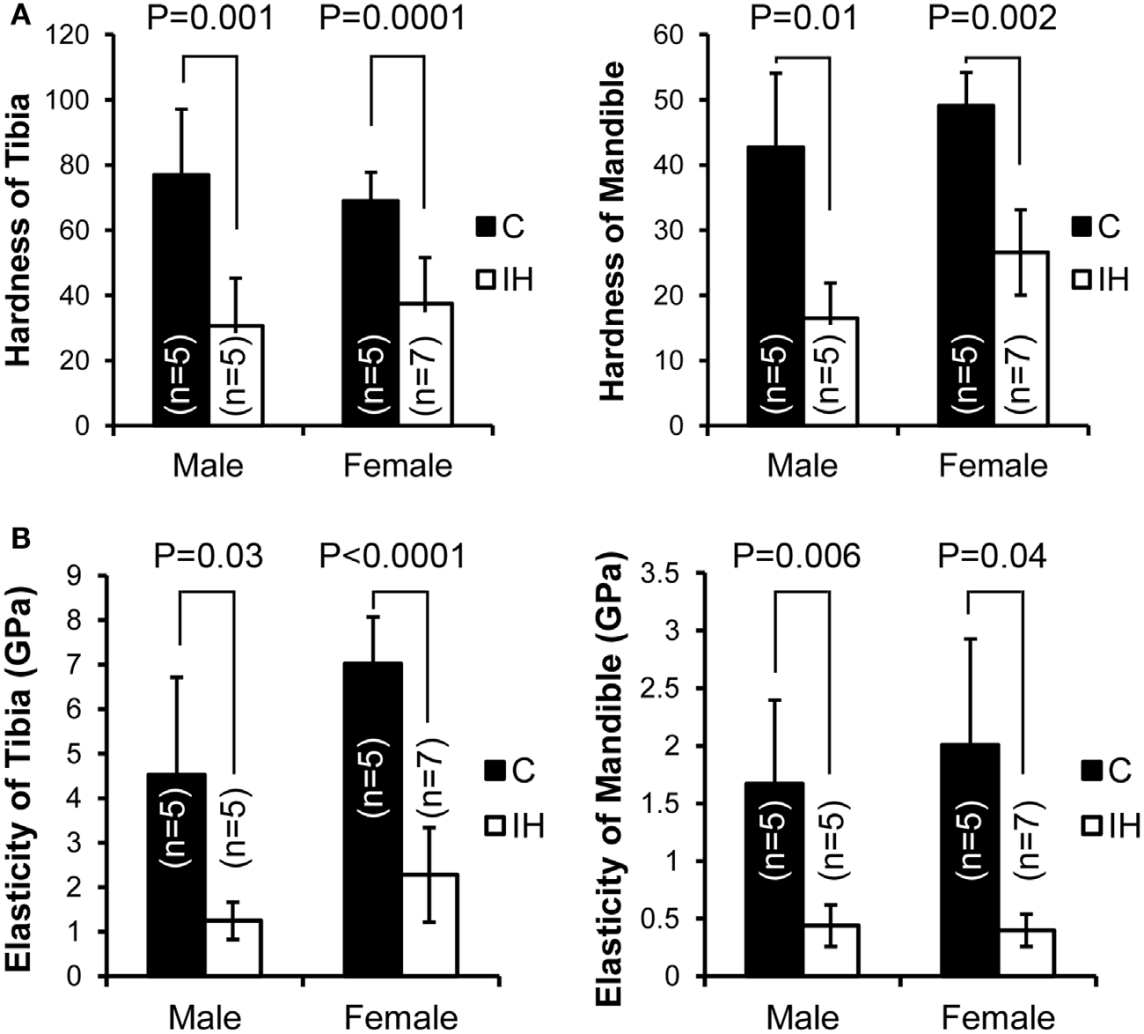
**Comparisons of dynamic hardness, and elasticity, of a long bone (tibia) and the mandible between male and female control and IH animals**. C (closed) represents the control group (five males and five females), and IH (open) represents the intermittent hypoxia group (five males and seven females). **(A)** Note that hardness in males (control tibia 76.9 ± 20.20 vs. IH tibia 30.5 ± 14.78 and control mandible 42.6 ± 11.41 vs. IH mandible 16.5 ± 5.39) and in females (control tibia 68.9 ± 8.84 vs. IH tibia 37.4 ± 14.18 and control mandible 49.0 ± 5.11 vs. IH mandible 26.6 ± 6.57). **(B)** Note that elasticity of male control tibia 4.53 ± 2.19 vs. IH 1.25 ± 0.42 and control mandible values of 1.67 ± 0.73 vs. IH mandible 0.44 ± 0.18. Control female tibia values were 7.02 ± 1.05 vs. IH tibia 2.28 ± 1.06, and female control mandible values were 2.01 ± 1.92 vs. IH mandible of 0.40 ± 0.14.

Measurements, including DH and elastic modulus, were obtained from each load–displacement curve, and means and SDs were calculated (Figure 1). Both hardness and elasticity significantly differed between control and IH-treated tibias and mandibles in accordance with *t*-tests. DH in IH-treated animals was approximately 60% of control values in males. However, in females, the IH treatment effect was less pronounced, approximating 45% (Figure 2A). Differences between IH and control groups in elasticity were greater than those of DH for both males (approximately 70%) and females (approximately 67–80%) (Figure 2B). A notable further disparity (10% or more decline in elasticity compared to the declined value for hardness) appeared after IH treatment in both long bones and mandibles. The results suggest that a short-term neonatal exposure to IH damages bone integrity in rodents with sex-dependent manner on hardness and elasticity.

### Histological and Subcellular Changes of the Bones after IH Exposure

**Figure 3:**
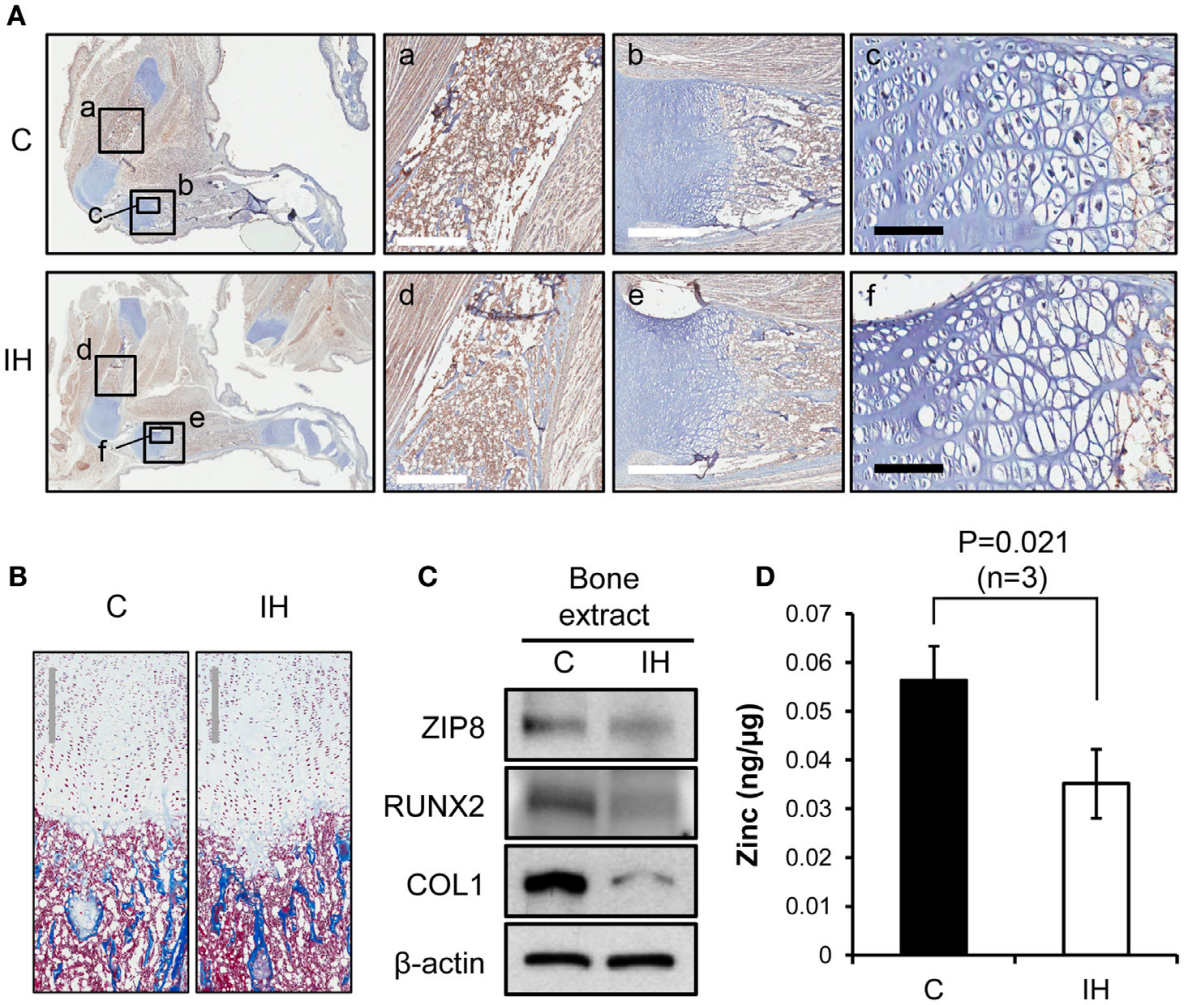
**Comparisons of bone integrity between controls and IH rats**. **(A)** First column: overview from which a–f panels are derived. Immunohistochemical staining using Runx2 antibody shows decreased expression of Runx2 protein (brown color) in the femur, harvested from IH-treated animals (panel d), over control (panel a). Panels b and e contrast the differences in the length of growth plate in the tibia (white scale bar = 500 μm). Panels c and f show cell density of the proliferating and hypertrophic zone of the growth plate (black scale bar = 100 μm). **(B)** Masson trichrome staining visualizes the zones of proliferation and hypertrophy of growth plate areas. No significant difference in cell counts appears between control and IH-treated bones. Gray scale bars = 300 μm. **(C)** Western blot assays demonstrate a decreased cellular expression of Runx2, Collagen-1 (COL1), and ZIP8 transporter proteins with respect to β-actin. **(D)** Zinc content in the tibia (whole bone extracts, *n* = 3) declined significantly after IH treatment.

Immunohistochemical staining using Runx2 antibody showed decreased expression of Runx2 protein (brown color) in the diaphysis of the femur (Insets a and d), harvested from IH-treated animals (Figure 3A). The differences in diameter and length of the bones between groups appeared insignificant. The quantity of osteoblasts expressing Runx2 protein in the medullary area did not show a notable disparity. In the insets b and e of Figure 3A, epiphyseal plate (growth plate) areas were also magnified and highlighted. Masson trichrome staining further clarified the zones of proliferation and hypertrophy of growth plate areas (Figure 3B); yet no significant difference was noted in the growth plate areas as well. Western blot results indicated decreased expression of Runx2, Collagen-1, and ZIP8 transporter proteins (Figure 3C). Zinc content in whole-cell extracts of the tibia declined significantly after IH treatment (Figure 3D).

### Changes in Subcellular Compositions of Human Osteoblasts

**Figure 4:**
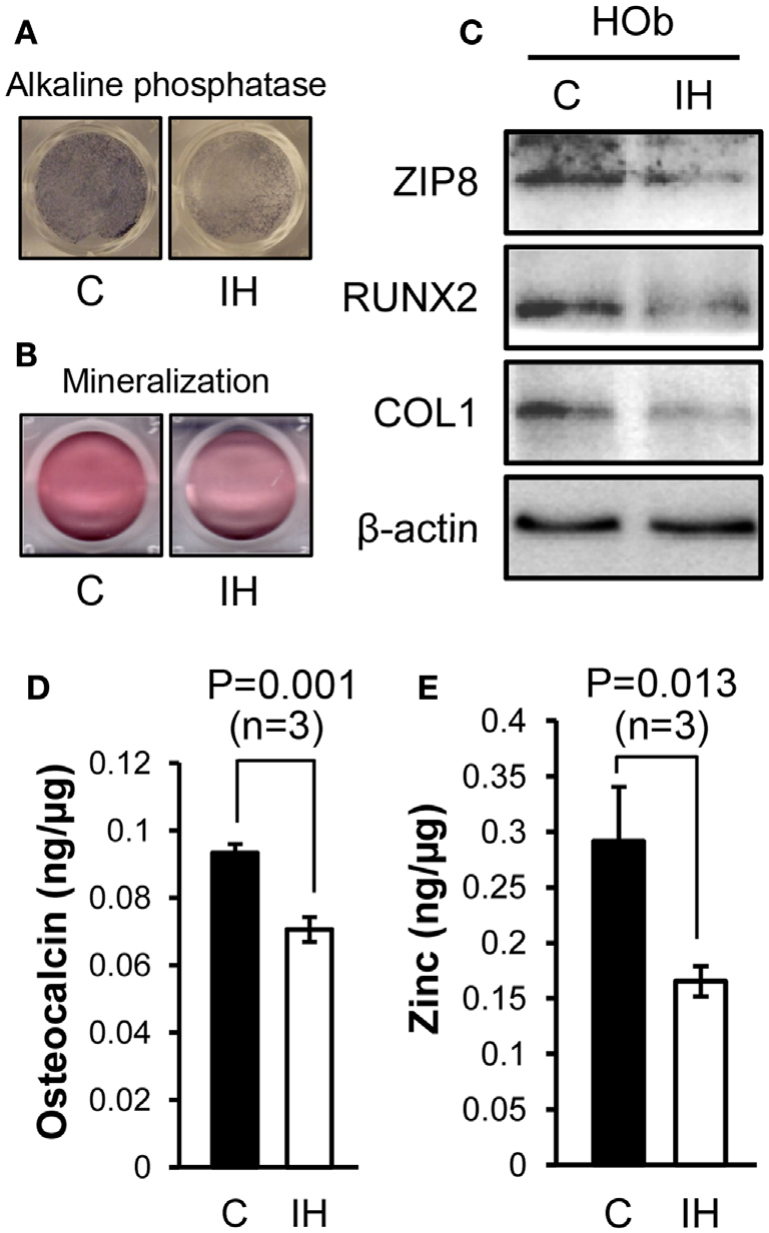
**Comparisons between control and IH-treated osteoblasts**. IH-treated human osteoblasts show decreased expression of alkaline phosphatase **(A)** and mineralization **(B)**, assessed by Alizarin S Red. Runx2, Collagen-1, and ZIP8 protein contents **(C)** assayed using Western blots. Osteocalcin levels **(D)** quantified from cell lysates using ELISA assays show a significant decline with IH. Zinc contents **(E)** estimated in whole-cell lysates obtained from three sets of osteoblasts culture dishes (*n* = 3), decreased after IH treatment.

After IH challenge, HObs showed decreased expression of alkaline phosphatase (Figure 4A) and mineralization (Figure 4B). Western blots noted decreased Runx2, Collagen-1, and ZIP8 protein contents (Figure 4C) as shown such in rat bone. Osteoclacin measurements shown in Figure 4D resulted in a significant decline after IH treatment. The amount of zinc estimated in whole-cell lysates obtained from three sets of osteoblasts culture (*n* = 3), significantly declined after IH treatment (Figure 4E). These results suggest that unbalanced zinc homeostasis may be a mechanism underlies a cause–effect relationship between IH insults and bone fragility.

### Zinc Effects on Bone Formation

**Figure 5:**
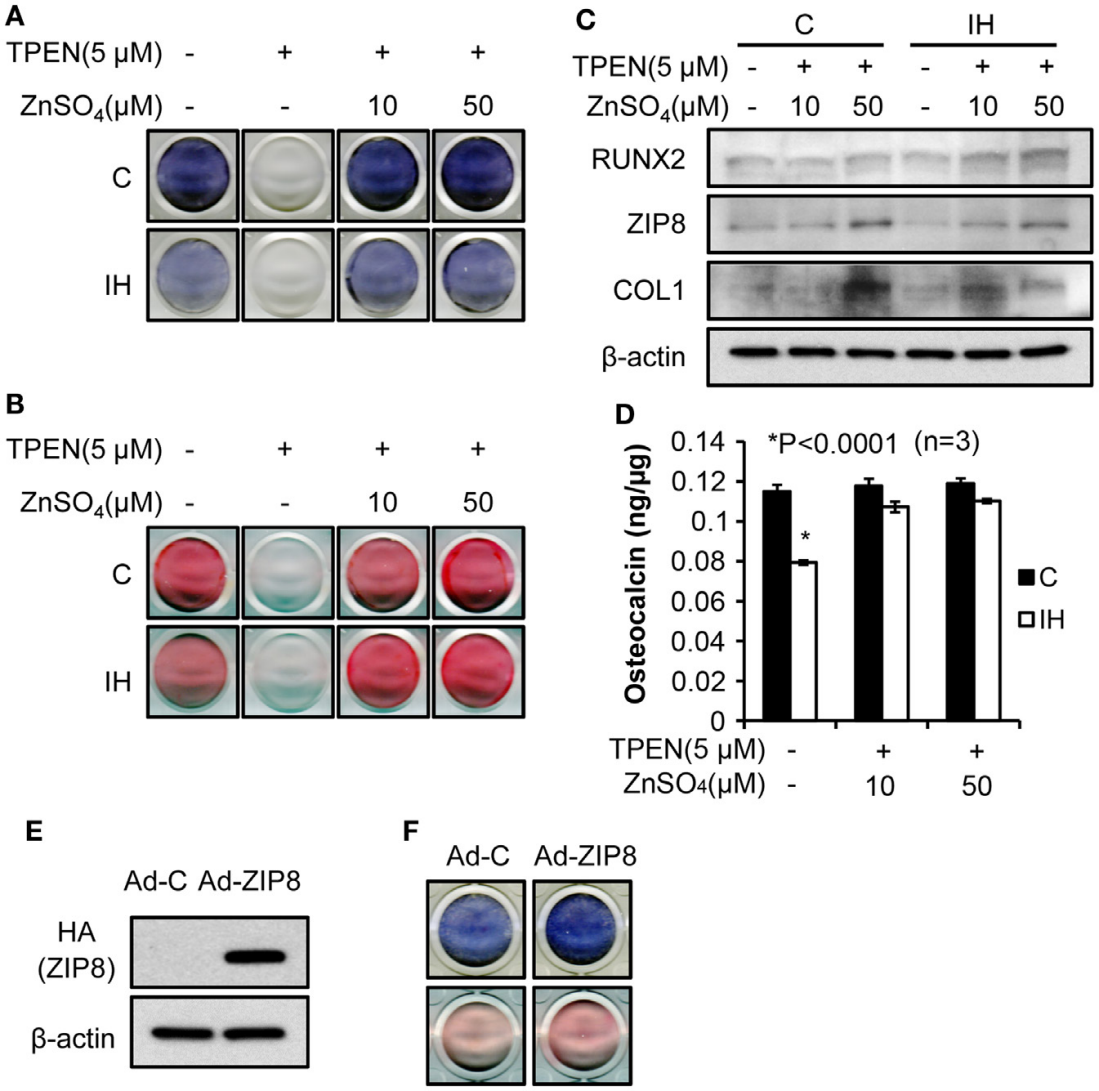
**Bone formation process rescued by zinc supplementation**. Alkaline phosphatase contents **(A)** and the degree of mineralization **(B)**, estimated by Alizarin S Red declined after IH treatment (control vs. IH in each column). After the zinc chelate TPEN was added, no signs of alkaline phosphatase **(A)** and mineralization **(B)** appeared [second column in **(A)** and **(B)**]. Supplementation by zinc sulfate recovers the expression of alkaline phosphatase and mineralization (+10 and +50 for IH). **(C)** Addition of zinc also increases the level of Runx2, ZIP8, and Collagen-1 protein expression (+10 and +50 for both control and IH). However, osteocalcin levels did not vary with zinc levels in the media **(D)** in control; yet, osteocalcin expression decreased under the IH condition significantly and recovered with zinc supplementation (see +10 and +50 for IH). **(E)** indicates that increased concentration of ZIP8 protein is of an exogenous origin, i.e., from adenovirus. **(F)** shows that ZIP8 transporter over-expression increased alkaline phosphatase production (top row in blue color) and bone mineralization (bottom row in pink color).

To investigate effects of zinc on the osteoblast differentiation after IH, the amount of alkaline phosphatase (Figure 5A) and the degree of mineralization (Figure 5B) were measured after application of TPEN (zinc chelate) or/and zinc supplement. Differentiation was declined by zinc chelate as shown on the second columns of Figures 5A,B. Yet, when zinc was added, the expression of alkaline phosphatase and the amount of mineralization significantly increased zinc concentration dependently. Western blot assays showed that the addition of zinc increases the level of Runx2, ZIP8, and Collagen-1 protein expression (Figure 5C). Osteocalcin levels measured in the control cells did not change along with the changes of zinc levels; yet the level of osteocalcin decreased significantly after IH treatment, then recovered by zinc addition (Figure 5D). When exogenous ZIP8 transporters were overexpressed using adenovirus (Figure 5E), expression of alkaline phosphatase and mineralization assessed by Alizarin S Red increased (Figure 5F). To recap, IH-damaged bone integrity could be reversed by zinc supplement.

### IH Effects on Glucose Regulation

Longer term IH effects were investigated for future direction on seven male pups. As shown in Table 1, the level of blood insulin were significantly low (176.3 vs. 15.3) at *p* = 0.001. The results suggest that IH effects on insulin excretion deteriorated further as animals age. Interestingly, however, blood glucose levels were maintained at a control level. No difference in weight was noted.

**Table 1:** **Insulin and glucose levels in blood of 12-week-old male rats had IH-treated immediate after birth**.

Sample size	Insulin (pM/L)	Glucose (mM/L)	Ratio (insulin/glucose)	Weight (g)
IH pups (*n* = 4)	15.3 ± 12.61*	9.1 ± 1.19	1.8 ± 1.50	494 ± 57
Control pups (*n* = 3)	176.3 ± 41.19*	9.3 ± 1.59	19.3 ± 5.77	490 ± 39

*p = 0.001.

## Discussion

The substantial declines in bone hardness and elasticity after brief neonatal IH exposure were unexpected. However, we already demonstrated that IH exposure elicits diabetes-like symptoms *via* a disturbed zinc homeostasis in pancreatic islets in rats (9); we investigated here whether a similar mechanism triggers a cause–effect loop between IH and an increased fragility of bones. Because apnea of prematurity is a common ventilation pattern in preterm neonatal babies, severe IH exposure could result in unexpected public health issue. Particularly, 12-week data set shown in Table 1 demands an extensive investigation on 12-week-old or older animals.

The intergroup differences in elasticity between IH-treated and control animals were greater than those of hardness, particularly in female pups. This finding may suggest a faulty composition of the extracellular matrix, which may be represented by the decreased Collagen-1 protein shown in Figure 3C. The *in vitro* studies showed a similar tendency. The extracellular matrix of bones is composed of collagen fibers, together with other fibers made of proteoglycan or elastin. Thus, the decreased collagen content and cross-link formation (23) in the extracellular matrix may be a critical factor that contributes to the significantly diminished bone elasticity in the tibia and mandible after IH exposure. The decreased osteocalcin levels after IH as shown in Figure 4D may underscore this explanation, because osteocalcin is a bone matrix protein that regulates hydroxyapatite size and shape through its vitamin-K-dependent, γ-carboxylated form (24, 25). More investigation of other proteins, in addition to Collagen-1 and osteocalcin, will clarify this issue.

We did not factor bone turnover processes in interpretation of these data; instead, we focused on bone formation, because bone formation is impaired in Type-1 diabetic patients (26). In our findings, a significant decrease in Runx2 protein in the IH group may well be expected, because Runx2 is a master transcription factor for osteogenesis, engaged in various up- and downstream functions of many proteins secreted by osteoblasts, which boost differentiation of osteoblasts (27). Regardless of the underlying condition, as Runx2 expression decreases, bone formation decreases (28). Yet, as discussed earlier by us and others, we do not know whether oxidative stress inflicted a decrease of Runx2 expression (29) or the decreased osteocalcin level influence Runx2 expression *via* a feedback process (30, 31).

Zinc is a trace metal involved in numerous essential body biochemical functions, including nucleic acid metabolism (32), and functions as a part of zinc finger proteins (33) and metalloenzymes for antioxidant function (34). Therefore, long-term zinc deprivation may result in serious dysfunction and structural damage in some organs, such as bone (35). Much evidence supports the finding that extracellular matrix mineralization is disturbed *via* Runx2 modulation by zinc (36–38). Zinc is also involved in calcium phosphate function (39) and leptin production (40), which helps modulate bone formation (25, 41). Thus, insufficient mineralization of bone spicule and matrix may be resulted from unbalanced activities of Runx2 and osteocalcin due to a sub-optimum level of cytosol zinc.

Zinc uptake transporters may play an important role in bone structure as well; zinc, metallothionein, and the interactions between the two are critical to bone formation (42). Zinc absorbed through diet should be imported into the cytoplasm of osteoblasts *via* zinc uptake transporters, such as ZIP8, which will boost osteocalcin production as well as bone formation after IH exposure. Despite ZIP8 transporter has been suggested to be a critical player in the pathogenesis of osteoarthritis (43), its functions in bone modeling and remodeling are largely unknown, because most research thus far has focused on zinc exporters (ZnT) (44).

A question arises whether zinc supplementation can reverse osteogenesis retardation in patients with osteoporosis (45), providing an inexpensive intervention for a costly condition. Our current findings support that possibility. Although the mechanisms associated with cytosolic zinc levels that can facilitate osteogenic, or suppress osteoclastic activities are unknown (46), research on various pathways is emerging (47, 48).

We did not solely aim to distinguish whether IH-induced neonatal diabetes results in decreased bone integrity or whether the increased bone fragility may result from a loss of zinc homeostasis. Further, it is not yet conclusive whether the findings here are secondary to neural disturbances, e.g., brain or ganglia structural damage resulting in sympathetic disturbances triggered by IH insults, or secondary to leptin dysfunction alone (49). Therefore, whether zinc deficiency is the primary mechanism for the reduced hardness and elasticity of bones deems to require further challenges. However, we speculate that zinc supplementation could help patients exposed to IH by such mechanisms as apnea of prematurity at an early age. It is unclear if zinc supplementation would reverse the progress of tissue damage or ongoing pathological changes in human bones; such a determination would require a controlled clinical trial partitioning both IH exposure and zinc levels.

## Author Contributions

All authors listed have made substantial, direct, and intellectual contribution to the work and approved it for publication.

## Conflict of Interest Statement

The authors declare that the research was conducted in the absence of any commercial or financial relationships that could be construed as a potential conflict of interest.
